# Clinical, radiological and pathological characteristics of moderate to fulminant psittacosis pneumonia

**DOI:** 10.1371/journal.pone.0270896

**Published:** 2022-07-11

**Authors:** Xiaoying Li, Tao Xiao, Pengzhi Hu, Kun Yan, Jiongxing Wu, Xinya Tu, Yishu Tang, Hong Xia

**Affiliations:** 1 Department of Emergency, The Third Xiangya Hospital, Central South University, Changsha, Hunan, China; 2 Department of Nursing, The Third Xiangya Hospital, Central South University, Changsha, Hunan, China; 3 Department of Radiology, The Third Xiangya Hospital, Central South University, Changsha, Hunan, China; University Campus Bio-Medico di Roma, ITALY

## Abstract

**Background:**

Psittacosis pneumonia is a community-acquired pneumonia caused by *Chlamydia psittaci*. It is usually under-diagnosed due to its atypical clinical presentation and lack of routine laboratory tests.

**Methods:**

To better understand the clinical features, 52 patients diagnosed with psittacosis pneumonia by metagenomic next-generation sequencing (mNGS) were enrolled in this study. The clinical, radiological and pathological characteristics were retrospectively analyzed.

**Results:**

The onset of psittacosis pneumonia in this study occurred all year round, with a peak from December to January. Most of the patients were 51–80 years old. About 65.38% of patients had a history of exposure to poultry or parrots. Abnormalities of multiple clinical signals were detected in these patients. Elevated levels of neutrophil ratio, C-reactive protein, erythrocyte sedimentation rate, and procalcitonin were detected in most patients. Radiological evidence revealed air-space consolidation or ground-glass opacities in lungs of all patients, which is the typical feature of psittacosis pneumonia. In addition, hyperemia, swelling of bronchial mucosa, and bronchial patency were detected by bronchoscopy in all patients, and bronchial sub-mucosal edema, inflammatory cells infiltration and alveolar epithelial hyperplasia were identified in the bronchial mucosa and alveolar tissue. Beta-lactam antibiotics were administered for empirical treatment before mNGS in 17 patients but showed no improvement. The treatment was switched to doxycycline or moxifloxacin immediately since psittacosis pneumonia were suspected and confirmed by mNGS detection (within 48 hours). After receiving adjustment of treatment, 94.23% (49/52) of patients were cured successfully.

**Conclusions:**

In conclusion, mNGS may be a promising approach for clinical diagnosis of psittacosis. For patients with a history of exposure to birds, hyperpyrexia, nonproductive cough, multiple elevated inflammatory markers, and air-space consolidation in lung, psittacosis pneumonia should be considered, especially when beta-lactam antibiotics showed limited efficacy.

## Background

Psittacosis, also termed as parrot fever, is a zoonotic disease caused by an obligate intracellular bacterium, *Chlamydia psittaci*. Birds (including parrots, poultry, pigeons, and other ornamental birds) and even mammals are the reservoirs of *C*. *psittaci* [1]. Direct contact with infected birds and mammals, exposure to their feces and respiratory secretions, and inhalation of contaminated aerosols may lead to human psittacosis [2, 3]. Person-to-person transmissions are rare [3].

A sudden attack of fever, nonproductive cough, headache, and myalgia are common in psittacosis patients. However, different patients with psittacosis may exhibit various clinical manifestations, including subclinical or influenza-like symptoms (mild psittacosis), pneumonia (moderate psittacosis), severe pneumonia (severe psittacosis), and severe pneumonia complicated with multiple organ dysfunction syndrome (MODS, fulminant psittacosis) [3–5].

Psittacosis pneumonia is generally under-diagnosed due to its atypical clinical presentation and lack of routine laboratory tests for *C*. *psittaci* [1, 2]. It can occur in any age group, principally between 40 and 64 years old, with an incubation period of 5–14 days, accounting for about 1.03% of patients with community-acquired pneumonia (CAP) [3, 6].

Serological methods, polymerase chain reaction (PCR) assays, and culture are the conventional diagnostic routines for psittacosis. However, there are many limitations. Serological methods often have a low specificity and time consuming. The sensitivity of PCR is relatively high, but it needs a prior hypothesis of the target [5, 7]. Isolation of *C*. *psittaci* can only performed in special laboratory due to biosafety concerns and complex procedure. So far, there is no gold standard protocol for the identification of human psittacosis [7]. Metagenomic next-generation sequencing (mNGS) is proposed as a promising approach for precise diagnosis of complicated infectious diseases in recent years, but the cost is relatively high [8–10].

Here, we describe the general, clinical, laboratorial, radiological, bronchoscopic, and pathological characteristics of psittacosis pneumonia diagnosed by mNGS in 52 patients. We aim to better understand the clinical features of psittacosis pneumonia, assisting the clinical identification of this disease. To our knowledge, this study represents the largest number of patients with severe to fulminant psittacosis pneumonia.

## Methods

### Study design and ethic statement

We retrospectively analyzed 52 patients with moderate to fulminant psittacosis pneumonia from the Third Xiangya hospital, central south university (a tertiary hospital) in Changsha, China, between June 2019 and October 2021. The baseline information, clinical symptoms and signs, laboratory testing results, radiological data, therapeutic schedules, and outcomes of these patients were obtained.

The informed consent was waived due to the retrospective study. The privacy and personally identifiable information of all subjects was protected. This study was approved by the Institutional Review Board of the Third Xiangya Hospital, Central South University, in compliance with the Declaration of Helsinki (as revised in 2013).

### Sampling and mNGS sequencing

Plasma samples were centrifuged from blood (3–4 ml) of patients. Bronchoalveolar lavage fluid (BALF) samples (0.5–3 ml) were obtained from patients by bronchoscopy. All samples were immediately sent to Hugobiotech, Co., Ltd. (Beijing, China) for PACEseq mNGS according to the standard work flow. In brief, DNA samples were extracted using TIANMicrobe Magnetic Patho-DNA/RNA Kit (Tiangen Biotech, Beijing, China) according to the manufacturer’s instructions. DNA libraries were constructed using QIAseqTM Ultralow Input Library Kit (Qiagen, Hilden, Germany), and the qualities were evaluated using Agilent 2100 Bioanalyzer (Agilent Technologies, Santa Clara, USA). Qualified DNA libraries were sequenced on the Illumina Nextseq 550Dx platform.

### Bioinformatic analysis

Adapters, low-quality, short (length <35 bp), and low-complexity reads were removed from the raw data. Human host sequences were filtered out by aligning the reads to human reference genome (hg38) using BWA software [11]. Finally, the reads left were aligned to the Microbial Genome Databases from National Center Biotechnology Information (fttp://ftp.ncbi.nlm.nih.gov/genomes/) employing BWA software.

### Diagnostic criteria

Diagnostic criteria for psittacosis pneumonia were as follows: (1) meeting the diagnostic criteria for CAP or severe CAP [12], (2) specific reads of *C*. *psittaci* genome were detected in BALF or blood samples via mNGS, (3) other etiologies were ruled out by routine diagnostic methods, including culture of blood, sputum, and BALF [4].

### Statistical analysis

The enumeration data were analyzed by Chi-square test or Fisher’s exact test. Continuous variables were displayed as means ± standard deviation for Gaussian distribution by T test or Chi-square test, or as medians (25^th^, 75^th^ percentiles) for non-Gaussian distribution by rank sum test. P<0.05 was deemed to reach statistical significance, and all statistical analysis were conducted by SPSS software (version 25.0, SPSS Inc, Chicago).

## Results

### General characteristics

A total of 52 patients, including 33 men and 19 women, were confirmed with *C*. *psittaci* infection by the above criteria, and 27 were diagnosed with severe to fulminant psittacosis pneumonia. The admission dates of these patients with psittacosis pneumonia peaked on December and January (S1 Fig). The age of patients ranged from 27 to 80 years, with a mean age of 61 years old. Most patients were 51–80 years old (S2 Fig).

About 65.38% (34/52) of patients had a history of exposure to poultry (including chickens, ducks, and geese) or pet parrots. In addition, one patient denied a history of exposure to birds or animals, but had contact with another confirmed patient 20 days before admission, suggesting a person-to-person transmission. Most patients were admitted to our hospital 7–8 days after onset, ranging from 2 to 30 days.

Approximately 61.54% of the patients suffered from chronic underlying diseases, including diabetes, hypertension, coronary heart disease, various cancers, cerebral infarction, *etc*. The general characteristics of these patients were summarized in S1 Table.

### Clinical characteristics

Fever (100%), cough (67.31%), fatigue (48.08%), dyspnea (44.23%), and chills (38.46%) were the common symptoms in these patients with psittacosis pneumonia. All patients complained of fever (38.5–40.4°C, mostly 39.0–40.0°C). The cough is mostly nonproductive cough, occasionally with a small amount of white sputum. Headache, abdominal pain or diarrhea, chest tightness or pain, myalgia, hemoptysis or blood-stained sputum, vomiting or loss of appetite, sore throat, nasal congestion and/or runny nose, convulsion or coma, and large tracts of erythema were also observed in some patients. The symptoms of psittacosis pneumonia were summarized in Table 1 and S2 Table. Increased respiratory rates (RR), decreased pulse oxygen saturation (SO2), relatively slow pulse rates, and moist and/or dry rales in the lungs were also obtained in some patients (Table 2).

**Table 1:** Clinical and radiologic characteristics of patients with psittacosis pneumonia.

Characteristics of patients	Abnormal patients / total patients
Male	63.46% (33/52)
Onset of age in group 51–80 years	84.62% (44/52)
Onset in December and January	42.31% (22/52)
Exposure history	67.31% (35/52)
Poultry	63.46% (33/52)
Parrots	1.92% (1/52)
Patients	1.92% (1/52)
Underlying diseases	61.54% (32/52)
Reads number of *Chlamydia psittaci*	2–270,670
**Symptoms**	
Fever	100% (52/52)
Cough	67.31% (35/52)
Fatigue	48.08% (25/52)
Dyspnea	44.23% (23/52)
Chills	38.46% (20/52)
Headache	19.23% (10/52)
Abdominal pain, diarrhea	19.23% (10/52)
Chest tightness or pain	15.38% (8/52)
Myalgia	15.38% (8/52)
Hemoptysis or blood-stained sputum	13.46% (7/52)
Vomiting or loss of appetite	11.54% (6/52)
Sore throat, nasal congestion and/or runny nose	7.69% (4/52)
Convulsion or coma	7.69% (4/52)
Large tracts of erythema	1.92% (1/52)
**Radiologic**	
Involved with multiple lobes	71.15% (37/52)
Involved with both lungs	57.69% (30/52)
Pleural effusion	51.92% (27/52)
Pericardial effusion	11.54% (6/52)
Severe lesions in the right upper lung	25.00% (13/52)
Severe lesions in the right lower lung	34.62% (18/52)
Severe lesions in the left upper lung	15.38% (8/52)
Severe lesions in the left lower lung	11.54% (6/52)
Severe lesions in the lower both lungs	13.46% (7/52)
Severe lesions in right lung	59.62% (31/52)
Severe lesions in left lung	26.92% (14/52)
Severe lesions in the upper lungs	40.38% (21/52)
Severe lesions in the lower lungs	59.62% (31/52)
**Treatment**	
Doxycycline or moxifloxacin	100% (52/52)
Noninvasive or invasive ventilation	48.08% (25/52)
Extracorporeal membrane oxygenation support	7.69% (4/52)
Hemofiltration therapy	3.85% (2/52)
Survive	94.23% (49/52)

**Table 2:** Signs and laboratory characteristics of patients with psittacosis pneumonia.

Characteristics	Abnormal patients / total patients	Range	Reference values
**Signs**			
Hyperpyrexia (°C)	100% (52/52)	38.5–40.4	36–37.2
Increased RR (times/min)	44.23% (23/52)	21–33	12–20
Decreased SO_2_ (%)	55.77% (29/52)	54–94	95–100
Relatively slow pulse rates (beats/min)	26.92% (14/52)	49–114	Increased 15–20 beats/min for every 1°C increase
Moist and/or dry rales	61.54% (32/52)	Unilateral lung or both lungs	No rales
**Laboratory**			
Decreased PaCO2 (mmHg)	90.48% (38/42)	16.6–34.5	35–45
Increased pH values	69.05% (29/42)	7.46–7.58	7.35–7.45
Decreased PaO2 or OI (mmHg)	78.57% (33/42)	PaO2 33.2–58.2; OI 64.4–298	PaO2 < 60 or OI <300
Increased WBC counts (×10^9^/L)	38.46% (20/52)	9.84–24.91	3.5–9.5
Decreased WBC counts (×10^9^/L)	9.61% (5/52)	2.72–3.39	3.5–9.5
Increased NE ratio (%)	86.54% (45/52)	76.3–96.5	40–75
Thrombocytopenia (×10^9^/L)	5.77% (3/52)	32–95	100–300
Increased CRP (mg/L)	100% (51/51)	6.34–369.77	0–6
Increased ESR (mm/hr)	97.62% (41/42)	32–120	0–28
Increased PCT (ng/ml)	97.83% (45/46)	0.07–60.01	0–0.05
Increased AST (U/L)	86.54% (45/52)	41–2009	15–40
Increased ALT (U/L)	75.00% (39/52)	61–846	9–50
Increased TBIL (μmol/L)	28.85% (15/52)	20.7–337.5	3.42–20.5
Increased DBIL (μmol/L)	46.15% (24/52)	6.9–261.1	0–6.84
Hypoalbuminemia (g/L)	90.38%(47/52)	17.2–33.8	<35
Increased BUN (mmol/L)	19.23% (10/52)	11.21–37.26	3.1–9.5
Increased CRE (μmol/L)	19.23% (10/52)	120–971	57–111
Elevated CK (U/L)	39.58% (19/48)	212–35530	40–200
Hyponatremia (mmol/L)	40.38% (21/52)	114.3–134.9	<135
Hypokalemia (mmol/L)	34.62% (18/52)	2.06–3.47	<3.5

ALT, alanine aminotransferase; AST, aspartate aminotransferase; BUN, blood urea nitrogen; CK, creatine kinase; CRE, creatinine; CRP, C-reactive protein; DBIL, direct bilirubin; ESR, erythrocyte sedimentation rate; NE, neutrophil ratio; PaCO2, arterial partial pressure of carbon dioxide; PaO2, arterial partial pressure of oxygen; PCT, procalcitonin; pH, pondus hydrogenii; RR, respiratory rates; TBIL, total bilirubin; WBC, white blood cell; SO2, oxygen saturation

### Laboratorial characteristics

Arterial blood gas (ABG) analysis was performed in 42 patients. Reduced arterial partial pressure of carbon dioxide (PaCO_2_) in 90.48% of patients, increased pondus hydrogenii values (pH) in 69.05% of patients, and decreased arterial partial pressure of oxygen (PaO_2_) or oxygenation index (OI) below 300 mmHg in 78.57% of patients were identified in this study.

White blood cell counts (WBC) were variable, and might decrease, be normal, or increase. Elevated neutrophil ratio (NE), C-reactive protein (CRP) levels, erythrocyte sedimentation rate (ESR), and procalcitonin (PCT) were detected in most patients (Table 2).

Hypoalbuminemia, as well as varying degrees of elevation of aspartate aminotransferase (AST) and alanine aminotransferase (ALT) were discovered in most patients. In total, 7 patients were diagnosed with multiple organ dysfunction syndrome, characterized by elevated AST, ALT, total bilirubin (TBIL), direct bilirubin (DBIL), blood urea nitrogen (BUN), creatinine (CRE), and 5 patients presented with rhabdomyolysis and significantly elevated creatine kinase (CK). The laboratory characteristics were described in Table 2 and S3 Table.

### Radiologic characteristic

**Fig 1:**
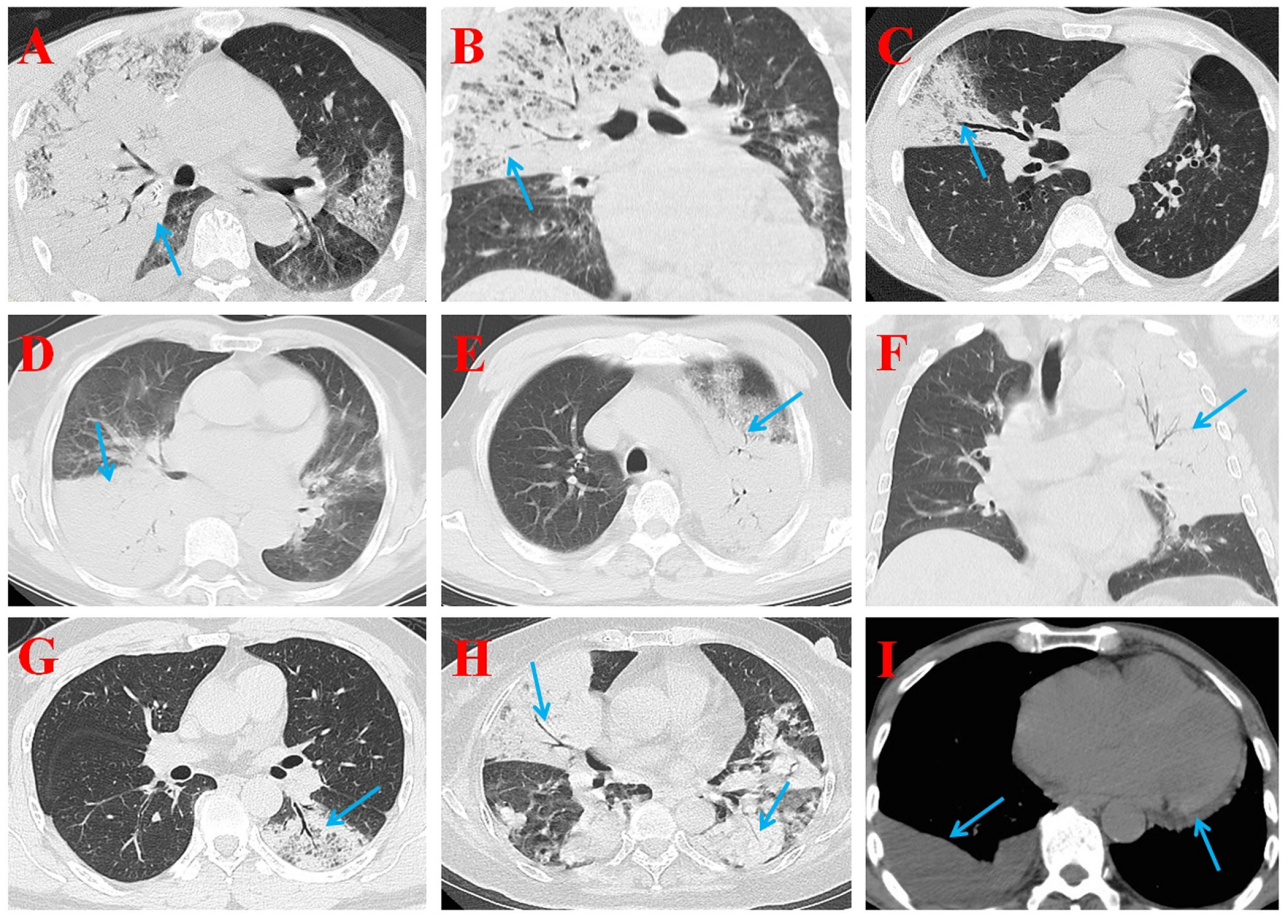
Radiologic characteristic of psittacosis pneumonia. Large consolidation or ground-glass opacities with air bronchial shadow were mostly identified in the right upper lobe (**A**, **B**), the right middle lobe (**C**), the right lower lobe (**D**), the left upper lobe (**E**, **F**), the left lower lobe (**G**), and the lower lobes of both lungs (**H**). The arrows in (**I**), indicated right pleural effusion and mild pericardial effusion.

Air-space consolidation or ground-glass opacities, and large areas of inflammation exudations were observed in lungs of all patients with psittacosis pneumonia (Fig 1). Pneumonia involving multiple lobes (at least two lobes) or both lungs was presented in most of patients. The radiologic characteristics were shown in Table 1 and Fig 1. Mild to moderate pleural effusion on one or both sides was detected in 51.92% of patients, and mild pericardial effusion was documented in 11.54% of patients. The lesions might be progressed to the peripheral and contralateral lobes [13].

### Bronchoscopic characteristic

**Fig 2:**
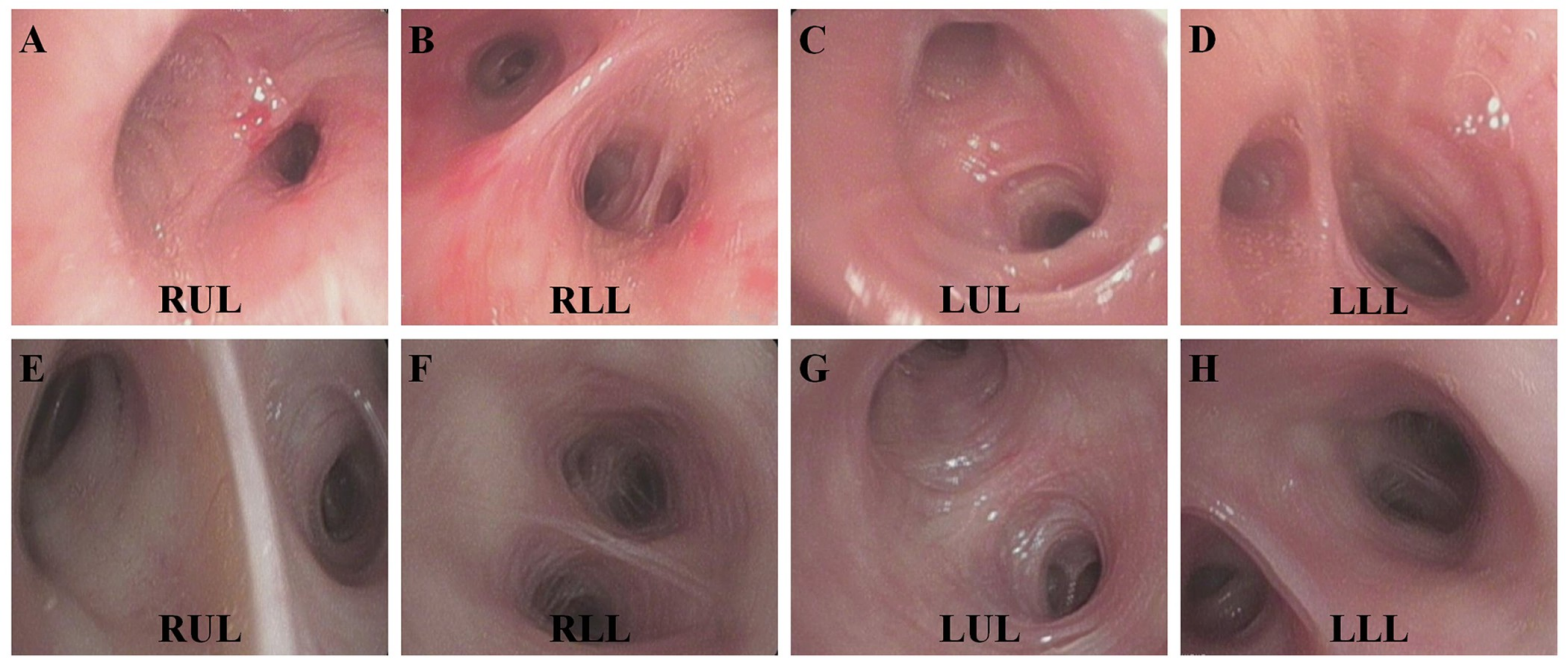
Bronchoscopic characteristic of psittacosis pneumonias. Mild hyperemia and swelling of bronchial mucosa, a little of white secretions, and patency of grade 1–4 bronchi in bilateral lungs were displayed in a patient (**A**, **B**, **C**, **D**) with psittacosis pneumonias in the right upper lobe and another patient (**E**, **F**, **G**, **H**) with the left upper lobe lesions by bronchoscopy, respectively. RUL, right upper lobe; LUL, left upper lobe; LLL, left lower lobe; RLL, right lower lobe.

Mild hyperemia and swelling of bronchial mucosa, a little of white secretions, and patency of grade 1–4 bronchi in bilateral lungs were the characteristics of most patients with psittacosis pneumonia by bronchoscopy (Fig 2). Scattered bronchial mucosal hemorrhage was occasionally detected in few patients.

### Pathological characteristic

**Fig 3:**
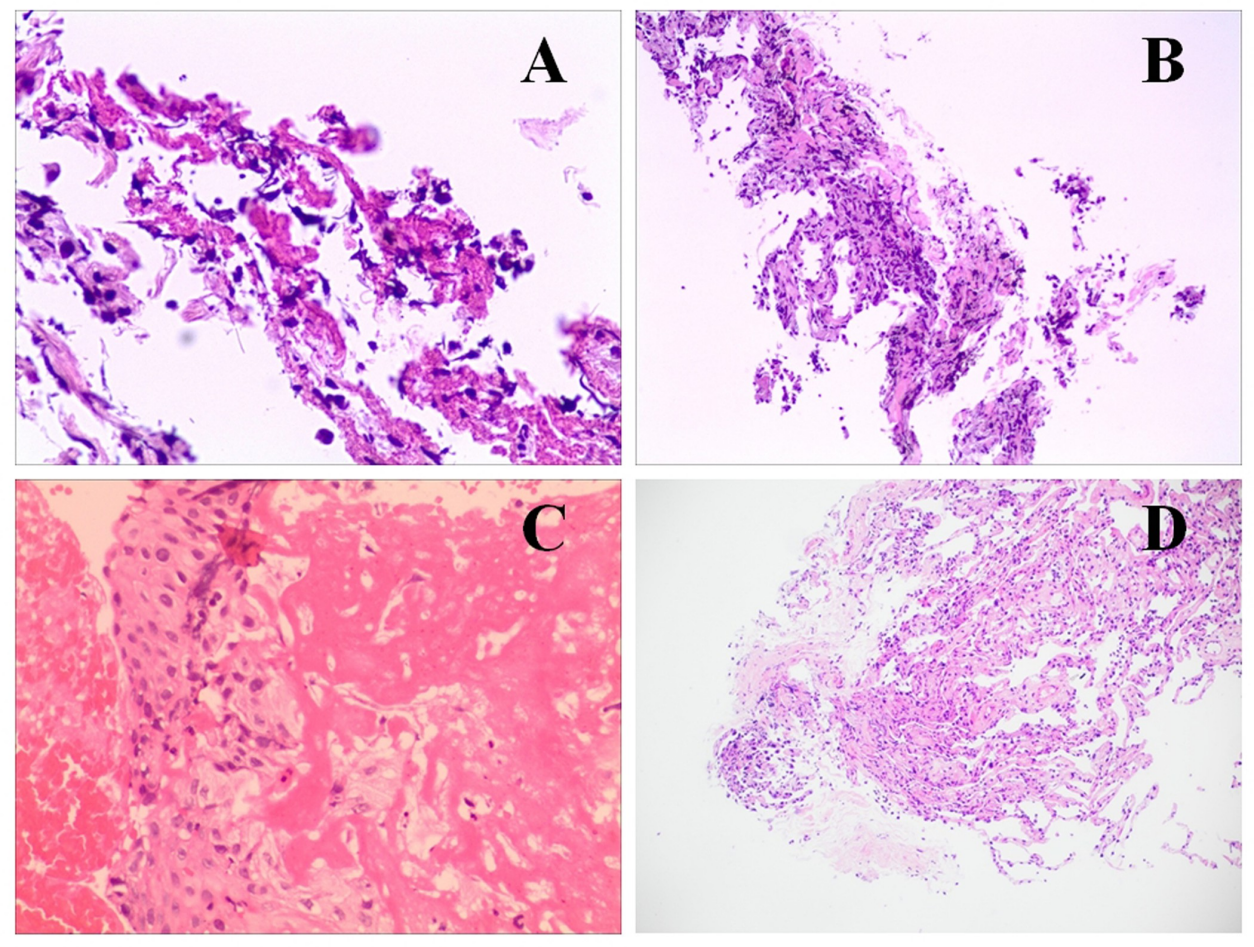
Pathological characteristic of psittacosis pneumonia. (**A**, **B**) Alveolar epithelial hyperplasia, interstitial edema, fiber hyperplasia, inflammatory cell infiltrations, some studdle substances or filaments in a few alveolar cavities were observed in the lung tissue. (**C**) Bronchial sub-mucosal edema, inflammatory cells infiltrations, and focal alveolar epithelial hyperplasia were identified in the bronchial mucosa and alveolar tissue. (**D**) Chronic changes, interstitial edema, and focal fibrinoid necrosis were discovered in the tracheal mucosal tissues from a convalescent patient.

Moderate amounts of tissue cells, as well as a small number of ciliated columnar epithelial cells, lymphocytes, and neutrophils were detected in some BALF samples. Bronchial sub-mucosal edema, little alveolar cavity opening, hemorrhage and cellulose exudations in most alveoli, histiocytic depositions, interstitial edema and fibrous hyperplasia, lymphocytes and neutrophil infiltrations, focal alveolar epithelial hyperplasia, focal myofibroblasts into the alveolar cavities in clumps, and some studdle substances or filaments in a few alveolar cavities were observed in the bronchial mucosa and alveolar tissues of some acute stage patients (Fig 3A–3C). Chronic changes, interstitial edema, and focal fibrinoid necrosis were discovered in the tracheal mucosal tissues from a convalescent patient (Fig 3D).

### Treatment and outcomes

Beta-lactam antibiotics were administered for empirical treatment before mNGS in 17 patients but showed no improvement. The treatment was switched to doxycycline or moxifloxacin immediately since psittacosis pneumonia were suspected and confirmed by mNGS detection (within 48 hours). Doxycycline (200 mg per day) or moxifloxacin (400 mg per day) were administered by intravenous drip for at least 14 days as recommended once psittacosis pneumonia was suspected or confirmed [3]. The symptoms of most patients generally improved, and the clinical signals returned to normal within several days after adjustment of treatments. Hypoalbuminemia, hyponatremia and hypokalemia were also gradually improved. Consolidations and infiltrates were gradually resolved within 10 days and completely resolved within six weeks or more.

In addition, 29 patients were admitted to intensive care unit, including one complicated with septic shock and respiratory failure who received post-cardiopulmonary resuscitation. Of the 29 patients, 24 had noninvasive or invasive ventilation, four received extracorporeal membrane oxygenation support, and two underwent hemofiltration therapy (Table 1 and S4 Table).

About 94.23% (49/52) of patients were cured successfully after doxycycline and moxifloxacin treatment, while three patients abandoned treatment due to severe respiratory failure and financial difficulty. Two patients were readmitted to our hospital due to psittacosis pneumonia within one year. Characteristics and statistical analysis of patients with psittacosis pneumonia were summarized in Table 3.

**Table 3:** Characteristics and statistical analysis of patients with psittacosis pneumonia.

Characteristics	Moderate group	Severe to fulminant group	t/χ^2^/Z	P value
Gender (M/F)	16/9	17/10	0.006	0.938
Age (years)	59 ± 9.64	63.04 ± 14.03	-1.217	0.230
Exposure history (Y/N)	15/10	21/6	1.926	0.125
Underlying diseases (Y/N)	11/14	21/6	6.257	**0.012**
Cough	15/25	20/27	1.169	0.280
Fatigue	12/25	13/27	-	0.605
Dyspnea	6/25	17/27	7.989	**0.005**
Chills	12/25	8/27	1.851	0.174
Headache	6/25	4/27	0.705	0.401
Abdominal pain, diarrhea	2/25	8/27	3.910	**0.048**
Chest tightness or pain	4/25	4/27	0.014	0.906
Myalgia	7/25	1/27	5.886	**0.015**
Hemoptysis or blood-stained sputum	4/25	3/27	0.266	0.606
Vomiting or loss of appetite	4/25	2/27	0.939	0.333
pH	7.52 (7.5, 7.54)	7.47 (7.41, 7.5)	-3.526	**<0.001**
OI (mmHg)	309.4 ± 12.89	197.59 ± 10.05	6.842	**<0.001**
PaCO_2_ (mmHg)	25.9 (23.7, 29)	28.8 (25.6, 31.6)	-1.470	0.141
WBC (×10^9^/L)	6.55 (5.03, 9.29)	9.84 (4.99, 15.19)	-1.456	0.145
NE (%)	84.4 (76.35, 90.1)	90.2 (86.6, 95.1)	-3.324	**0.001**
PLT (×10^9^/L)	245 (186, 350)	226 (130, 334)	-0.934	0.350
CRP (mg/L)	181.80 (80.17, 269.02)	233.08 (168.52, 274.94)	-1.438	0.151
ESR (mm/hr)	110 (86, 120)	82 (19.71, 120)	-1.301	0.193
PCT (ng/ml)	0.95 (0.25, 26.14)	4.1 (1.29, 18.04)	-1.639	0.101
AST (U/L)	67 (44, 107.5)	182 (94, 393)	-3.040	**0.002**
ALT (U/L)	76 (32, 105.5)	182 (94, 393)	-2.051	**0.040**
TBIL (μmol/L)	9.5 (6.1, 16.7)	112 (69, 265)	-3.169	**0.002**
DBIL (μmol/L)	3.7 (2.1, 7.05)	8.5 (5.1, 15.8)	-3.340	**0.001**
ALB (g/L)	26.9 (25.35, 33.05)	25.3 (22.1, 27.3)	-2.739	**0.006**
BUN (mmol/L)	4.79 (3.62, 6.28)	6.83 (5.36, 18.06)	-2.995	**0.003**
CRE (mmol/L)	72 (62, 84)	96 (63, 132)	-1.694	0.090
CK (U/L)	108 (74, 400)	160 (79, 351)	-0.330	0.742
Na (mmol/L)	137.7 (130.7, 140.35)	136.7 (133.1, 138.6)	-0.476	0.634
K (mmol/L)	3.66 (3.39, 4.27)	3.59 (3.08, 4.31)	-0.156	0.876
Diagnosis time after hospitalization (days)	3.52 ± 1.71	2.74 ± 1.38	1.801	0.075
Start time of specific treatment (days)	11.04 ± 6.37	8.74 ± 3.23	1.623	0.114
Length of stay (days)	9.44 ± 3.3	17.81 ± 9.94	-4.138	**<0.001**

ALB, albumin; ALT, alanine aminotransferase; AST, aspartate aminotransferase; BUN, blood urea nitrogen; CK, creatine kinase; CRE, creatinine; CRP, C-reactive protein; DBIL, direct bilirubin; ESR, erythrocyte sedimentation rate; F, female; K, potassium; M, male; Na, natrium; N, no; NE, neutrophil ratio; OI, oxygenation index; PaCO2, arterial partial pressure of carbon dioxide; PCT, procalcitonin; pH, pondus hydrogenii; PLT, platelet; TBIL, total bilirubin; t/χ^2^/Z, t test or χ^2^ test or Z test; WBC, white blood cell counts; Y, yes

## Discussion

In this study, we retrospectively analyzed the clinical, radiological, and pathological characteristics of psittacosis pneumonia in 52 patients. Most patients had a history of exposure to birds and complained of hyperpyrexia and nonproductive cough. For example, elevation of overall inflammatory markers (including NE, CRP, ESR and PCT) were detected in most patients. Radiological evidence revealed typical air-space consolidation or ground-glass opacities in lung of all patients. Empirical antibiotic therapy, sucha as beta-lactam antibiotics performed limited efficacy for psittacosis pneumonia. However, this disease was sensitive to doxycycline and moxifloxacin, and most patients can be completely cured after the targeted treatment.

There were more male than female of patients with psittacosis pneumonia in this study, in accord with the previous study [4]. This may be associated with occupational exposure, such as feeding or killing poultry. Though occurred all year round, psittacosis pneumonia is more common in winter, especially on December and January in China. This may be associated with reduced resistance to pathogens of patients and longer survival time of *C*. *psittaci* in the cold season [14]. The onset age mostly fell in 51 to 80 years old, which was higher than the previous report (40 to 64 years old) [3]. One reason was that there were more severe psittacosis pneumonia patients in the study, and elderly patients are more likely to have severe psittacosis pneumonia. Two pregnant women were diagnosed with severe psittacosis pneumonia, one with stillborn, this suggested gestational psittacosis may lead to stillborn or premature delivery [15].

Fever, cough, fatigue, dyspnea, chills, and headache were the common symptoms of psittacosis pneumonia, consistent with previous reports [3, 4]. Other symptoms including abdominal pain, diarrhea, chest tightness, myalgia, hemoptysis or blood-stained sputum, vomiting, loss of appetite, dysphoria, sore throat, nasal congestion, runny nose, convulsion, coma, and large tracts of erythema were also observed in a few patients in this study. These varied symptoms may lead to delayed diagnosis of psittacosis pneumonia.

Hyperpyrexia, mostly at 39.0–40.0°C, was the most common signs in patients with psittacosis pneumonia. Moist and dry rales in lungs, increased RR, and decreased SO2 were also the common signs. Relatively slow pulse rates (pulse-temperature dissociation) were also detected in some patients of this study, which consisted with in previous study [3, 16]. However, no obvious rales in the lungs were responsible for about 38.5% of patients with psittacosis pneumonia, resulting in absence of lung imaging, which may underestimate the extent of lesions in the lungs [3].

ABG is usually used for respiratory failure detection with good performance. Increased pH values and reduced PaCO2 and PaO2 may be associated with fever, increased RR, and severe pneumonia. Increased NE ratio, CRP, ESR, and PCT levels were discovered in most of patients, consistent with the previous study [17, 18], suggesting these inflammatory biomarkers may contribute to assessing the infection of *C*. *psittaci*. Varied degrees of elevation in AST, ALT, TBIL, DBIL, BUN, CRE, and CK levels were identified in patients with severe psittacosis pneumonia and MODS, including respiratory failure, liver dysfunction, renal dysfunction and/or rhabdomyolysis [19, 20].

Most patients presented with hypoalbuminemia in this study, which was not described in previous reports. This might be associated with a decrease in albumin intake due to loss of appetite, a decrease in albumin production due to hepatic injury, and an increase in albumin consumption due to hyperthermia. Hyponatremia and hypokalemia also occurred in some patients with psittacosis pneumonia, consistent with the previous study [17], this may be related to massive loss of blood sodium and potassium caused by high fever.

Air-space consolidation or ground-glass opacities, and large areas of inflammation exudation in one or more lobes were the characteristic radiographic changes in psittacosis pneumonia, in accord with the previous reports [4]. Either the superior lobes or inferior lobes may be the first to be infected.

Multiple lobes or both lungs were involved in most of patients with psittacosis pneumonia, the frequency was higher than the previous reports [4]. In fact, previous studies revealed pneumonia involving single lower lobar lesions was the most frequent in psittacosis pneumonia patients [13, 21].

Pleural effusions were detected in most of patients with severe psittacosis pneumonia, consistent with the previous study [4], and pericardial effusions were also discovered in some patients. In this study, the incidence of lesions in right lung (31/52, 59.62%) was higher than that in left lung (14/52, 26.92%), and the incidence in lower lungs was slightly higher than upper lungs. This might be related to the anatomical structure of left and right bronchus.

The radiographic features of psittacosis pneumonia in this study included air-space consolidation or ground-glass opacities and inflammation exudation of one or more lobes in single or both lungs, partly with pleural effusions and/or pericardial effusions. Lung lesions could occur in any lobe, especially on the right lung.

Mild hyperemia and swelling of bronchial mucosa, a little of white secretions, bronchial patency, and scattered bronchial mucosal hemorrhage were detected by bronchoscopy, in accord with the previous study [21].

Bronchial sub-mucosal edema, little alveolar cavity opening, hemorrhage and cellulose exudations in most alveoli, histiocytic depositions, interstitial edema and fibrous hyperplasia, lymphocytes and neutrophils infiltrations, and focal alveolar epithelial hyperplasia were the pathological features of psittacosis pneumonia during acute inflammation. Alveolar epithelial hyperplasia, interstitial edema, fiber hyperplasia, inflammatory cell infiltrations, and focal fibrinoid necrosis were the pathological characteristics of psittacosis pneumonia during convalescence. On account of the specific origin of the studdle substances or filaments in the alveolar cavities were not identified by special staining, it might be a specific pathological feature of C. *psittaci* pneumonia.

Progression and mortality of psittacosis pneumonia are closely associated with delay in diagnosis and treatment, and mNGS was a rapid and accurate approach for identification of *C*. *psittaci* [5]. In this study, mNGS successfully detected the pathogen in psittacosis pneumonia patients within 48 hours. This helped the timely adjustment of treatment for *C*. *psittaci*. Though half of the patients in this study suffered from severe to fulminant psittacosis pneumonia and experienced an even worse prognosis. The survival rate of psittacosis pneumonia in this study reached 94.23% (49/52), consistent with that (94.20%) of cumulatively reported cases during 2012 and 2020 [22].

## Conclusion

In conclusion, mNGS may be a potential approach for clinical diagnosis of psittacosis. For patients with a history of exposure to birds, hyperpyrexia, nonproductive cough, multiple elevated inflammatory markers, and air-space consolidation in lung, psittacosis pneumonia should be considered, especially when beta-lactam antibiotics showed limited efficacy.

## Supporting information

S1 Table General characteristics of the patients with psittacosis pneumonia. (DOCX)

S2 Table Clinical characteristics of the patients with psittacosis pneumonia. (DOC)

S3 Table Laboratory characteristics of the patients with psittacosis pneumonia. (DOC)

S4 Table Radiologic characteristics, treatment and outcomes of the patients with psittacosis pneumonia. (DOC)

S1 Fig The distribution of admission cases. (TIF)

S2 Fig The number of cases in different age groups. (TIF)

## Abbreviations

ALT: alanine aminotransferase
AST: aspartate aminotransferase
BALF: bronchoalveolar lavage fluid
BUN: blood urea nitrogen
CAP: community-acquired pneumonia
CK: creatine kinase
CRE: creatinine
CRP: C-reactive protein
DBIL: direct bilirubin
ESR: erythrocyte sedimentation rate
mNGS: metagenomic next-generation sequencing
NE: neutrophil ratio
PaCO2: arterial partial pressure of carbon dioxide
PaO2: arterial partial pressure of oxygen
PCR: polymerase chain reaction
PCT: procalcitonin
pH: pondus hydrogenii
RR: respiratory rates
TBIL: total bilirubin
WBC: white blood cell
SO2: oxygen saturation

## References

[1] Hogerwerf L, Roof I, de Jong MJK, Dijkstra F, van der Hoek W. Animal sources for zoonotic transmission of psittacosis: a systematic review. *BMC Infect Dis*. 2020; 20(1):192. doi: 10.1186/s12879-020-4918-y 32131753 PMC7057575

[2] Gu L, Liu W, Ru M, Lin J, Yu G, Ye J, et al. The application of metagenomic next-generation sequencing in diagnosing Chlamydia psittaci pneumonia: a report of five cases. *BMC Pulm Med*. 2020; 20(1):65. doi: 10.1186/s12890-020-1098-x 32178660 PMC7077129

[3] Balsamo G, Maxted AM, Midla JW, Murphy JM, Wohrle R, Edling TM, et al. Compendium of measures to control Chlamydia psittaci infection among humans (psittacosis) and pet birds (avian chlamydiosis), 2017. *J Avian Med Surg*. 2017; 31(3):262–282. doi: 10.1647/217-265 . 28891690

[4] Su S, Su X, Zhou L, Lin P, Chen J, Chen C, et al. Severe Chlamydia psittaci pneumonia: clinical characteristics and risk factors. *Ann Palliat Med*. 2021; 10:8051–8060. doi: 10.21037/apm-21-1502 34353090

[5] Wang K, Liu X, Liu H, Li P, Lin Y, Yin D, et al. Metagenomic diagnosis of severe psittacosis using multiple sequencing platforms. *BMC Genomics*. 2021; 22(1):406. doi: 10.1186/s12864-021-07725-9 34078288 PMC8173916

[6] Hogerwerf L DE Gier B, Baan B, VAN DER Hoek W. Chlamydia psittaci (psittacosis) as a cause of community-acquired pneumonia: a systematic review and meta-analysis. *Epidemiol Infect*. 2017; 145(15):3096–3105. doi: 10.1017/S0950268817002060 28946931 PMC9148753

[7] Nieuwenhuizen AA, Dijkstra F, Notermans DW, van der Hoek W. Laboratory methods for case finding in human psittacosis outbreaks: a systematic review. *BMC Infect Dis*. 2018; 18(1):442. doi: 10.1186/s12879-018-3317-0 30165831 PMC6118005

[8] Miao Q, Ma Y, Wang Q, et al. Microbiological diagnostic performance of metagenomic next-generation sequencing when applied to clinical practice. *Clin Infect Dis*. 2018; 67(suppl_2):S231–S240. doi: 10.1093/cid/ciy693 30423048

[9] Gu W, Deng X, Lee M, Sucu YD, Arevalo S, Stryke D, et al. Rapid pathogen detection by metagenomic next-generation sequencing of infected body fluids. *Nat Med*. 2021; 27(1):115–124. doi: 10.1038/s41591-020-1105-z 33169017 PMC9020267

[10] Chiu CY, Miller SA. Clinical metagenomics. *Nat Rev Genet*. 2019; 20(6):341–355. doi: 10.1038/s41576-019-0113-7 30918369 PMC6858796

[11] Li Heng. Aligning sequence reads, clone sequences and assembly contigs with BWA-MEM. arXiv 2013:1303.3997v2 [q-bio.GN]

[12] Mandell LA, Wunderink RG, Anzueto A, Bartlett JG, Campbell GD, Dean NC, et al. Infectious Diseases Society of America/American Thoracic Society consensus guidelines on the management of community-acquired pneumonia in adults. *Clin Infect Dis*. 2007; 44(Suppl 2):S27–S72. doi: 10.1086/511159 17278083 PMC7107997

[13] Kong CY, Zhu J, Lu JJ, Xu ZH. Clinical characteristics of Chlamydia psittaci pneumonia. *Chin Med J (Engl)*. 2021; 134(3):353–355. doi: 10.1097/CM9.0000000000001313 33410632 PMC7846496

[14] Rodolakis A. In vitro and in vivo properties of chemically induced temperature-sensitive mutants of Chlamydia psittaci var. ovis: screening in a murine model. *Infect Immun*. 1983; 42(2):525–530. doi: 10.1128/iai.42.2.525-530.1983 6642642 PMC264460

[15] Paul L, Comstock J, Edes K, Schlaberg R. Gestational psittacosis resulting in neonatal death identified by next-generation RNA sequencing of postmortem, formalin-fixed lung tissue. *Open Forum Infect Dis*. 2018; 5 (8):ofy172. doi: 10.1093/ofid/ofy172 30151406 PMC6105100

[16] Fukui S, Kawamura W, Uehara Y, Naito T. A patient with psittacosis from a pigeon: A reminder of the importance of detailed interviews and relative bradycardia. *IDCases*. 2021; 25:e01164. doi: 10.1016/j.idcr.2021.e01164 34040985 PMC8142253

[17] Chen X, Cao K, Wei Y, Qian Y, Liang J, Dong D, et al. Metagenomic next-generation sequencing in the diagnosis of severe pneumonias caused by Chlamydia psittaci. *Infection*. 2020; 48(4):535–542. doi: 10.1007/s15010-020-01429-0 32314307 PMC7223968

[18] Wu HH, Feng LF, Fang SY. Application of metagenomic next-generation sequencing in the diagnosis of severe pneumonia caused by Chlamydia psittaci. *BMC Pulm Med*. 2021; 21(1):300. doi: 10.1186/s12890-021-01673-6 34556069 PMC8461849

[19] Meijer R, van Biezen P, Prins G, Boiten HJ. Multi-organ failure with necrotic skin lesions due to infection with Chlamydia psittaci. *Int J Infect Dis*. 2021; 106:262–264. doi: 10.1016/j.ijid.2021.03.091 33823280

[20] Zhang H, Zhan D, Chen D, Huang W, Yu M, Li Q, et al. Next-generation sequencing diagnosis of severe pneumonia from fulminant psittacosis with multiple organ failure: a case report and literature review. *Ann Transl Med*. 2020; 8 (6):401. doi: 10.21037/atm.2020.03.17 32355845 PMC7186658

[21] Li N, Li S, Tan W, Wang H, Xu H, Wang D. Metagenomic next-generation sequencing in the family outbreak of psittacosis: the first reported family outbreak of psittacosis in China under COVID-19. Emerg Microbes Infect. 2021; 10(1):1418–1428. doi: 10.1080/22221751.2021.1948358 34176434 PMC8284143

[22] Teng XQ, Gong WC, Qi TT, Li GH, Qu Q, Lu Q, et al. Clinical analysis of metagenomic next-generation sequencing confirmed Chlamydia psittaci pneumonia: A case series and literature review. *Infect Drug Resist*. 2021; 14:1481–92. doi: 10.2147/IDR.S305790 33888999 PMC8057788

