# Supplementary material for: Clinical, radiological and pathological characteristics of moderate to fulminant psittacosis pneumonia
Source: PLoS One. 2022 Jul 11;17(7):e0270896. doi: 10.1371/journal.pone.0270896 (PMC9273088; doi:10.1371/journal.pone.0270896)
Supplement: S1 Table — (DOCX) [file pone.0270896.s001.docx]

Supplementary Table 1 General characteristics of the patients with psittacosis pneumonia

| Cases | Gender | Age  (years) | Admission month | Exposure history | Symptom durations before admission (days) | Underlying diseases or others |
| --- | --- | --- | --- | --- | --- | --- |
| 1# | M | 69 | August | Poultry raising | 20 | NA |
| 2# | M | 68 | July | NA | 4 | Duodenal papillary cancer |
| 3# | M | 65 | June | Poultry raising | 6 | Coronary heart disease, hypertension |
| 4# | M | 41 | January | NA | 6 | NA |
| 5# | F | 49 | January | NA | 7 | NA |
| 6# | F | 61 | January | Exposure to Case 19 | 30 | [Choledocholithotomy](dic://choledocholithotomy%20and%20t-tube%20drainage) |
| 7# | F | 48 | January | Poultry raising | 10 | NA |
| 8# | F | 41 | January | Exposure to poultry | 7 | Lithangiuria |
| 9# | M | 70 | March | NA | 8 | Hypertension |
| 10# | M | 73 | September | Poultry raising | 7 | NA |
| 11# | F | 61 | December | NA | 5 | Breast cancer |
| 12# | F | 64 | January | NA | 6 | NA |
| 13# | M | 52 | January | Exposure to poultry | 9 | Diabetes |
| 14# | M | 70 | September | Poultry raising | 12 | NA |
| 15# | M | 58 | January | Poultry raising | 3 | Diabetes, hypertension |
| 16# | F | 58 | September | Poultry raising | 10 | NA |
| 17# | M | 75 | October | Exposure to poultry | 7 | Hypertension |
| 18# | M | 68 | September | Poultry raising | 7 | NA |
| 19# | M | 56 | February | NA | 5 | NA |
| 20# | M | 61 | February | Poultry raising | 3 | NA |
| 21# | M | 45 | October | Exposure to poultry | 6 | Diabetes |
| 22# | F | 57 | October | NA | 10 | Uremia |
| 23# | M | 59 | January | NA | 15 | NA |
| 24# | M | 52 | March | NA | 9 | NA |
| 25# | F | 54 | September | Exposure to poultry | 7 | NA |
| 26* | F | 27 | August | Parrot raising | 10 | Gestation period |
| 27* | M | 78 | February | NA | 2 | Coronary heart disease, hypertension |
| 28* | M | 58 | January | Poultry raising | 7 | NA |
| 29* | M | 76 | September | NA | 8 | Diabetes, hypertension, prostate cancer |
| 30* | M | 71 | May | Poultry raising | 5 | Hypertension |
| 31* | F | 72 | December | Exposure to poultry | 7 | Hypertension |
| 32* | F | 69 | October | Poultry raising | 7 | Diabetes, hypertension |
| 33* | M | 78 | July | Poultry raising | 7 | Parkinson's disease |
| 34* | M | 52 | July | NA | 13 | NA |
| 35* | M | 61 | June | NA | 7 | Alcoholic liver disease, diabetes |
| 36* | F | 70 | December | NA | 6 | Cerebral infarction, diabetes, hypertension |
| 37* | M | 80 | December | Poultry raising | 7 | Hypertension, radical resection of rectal carcinoma |
| 38* | M | 51 | December | Poultry raising | 8 | Gastric ulcer |
| 39* | F | 56 | December | Poultry raising | 7 | NA |
| 40* | M | 78 | December | NA | 7 | Pulmonary tuberculosis |
| 41* | M | 76 | December | Poultry raising | 10 | Alzheimer's disease, auricular fibrillation, hepatitis |
| 42* | M | 67 | December | Poultry raising | 7 | Carcinoma of rectum, diabetes, hypertension |
| 43* | M | 57 | November | Exposure to poultry | 4 | Gout, pulmonary tuberculosis |
| 44* | F | 69 | November | Poultry raising | 7 | Diabetes, hypertension |
| 45* | M | 64 | October | Exposure to poultry | 5 | Hepatitis B, post-cardiopulmonary resuscitation, severe pancreatitis |
| 46* | M | 43 | December | Poultry [raising](javascript:;) | 6 | Diabetes, gall-stone |
| 47* | M | 61 | December | Poultry raising | 4 | Hypertension |
| 48* | F | 58 | November | Poultry raising | 24 | Diabetes |
| 49* | F | 61 | February | Exposure to poultry | 7 | NA |
| 50* | M | 64 | January | NA | 10 | Diabetes, hypertension |
| 51* | F | 77 | August | Exposure to poultry | 7 | Diabetes |
| 52* | F | 28 | November | Exposure to poultry | 18 | Stillborn at 16 weeks |

#patients with moderate psittacosis pneumonia; *patients with severe to fulminant psittacosis pneumonia

F, female; M, male; NA, not available
