# Supplementary material for: Clinical, radiological and pathological characteristics of moderate to fulminant psittacosis pneumonia
Source: PLoS One. 2022 Jul 11;17(7):e0270896. doi: 10.1371/journal.pone.0270896 (PMC9273088; doi:10.1371/journal.pone.0270896)
Supplement: S2 Table — (DOC) [file pone.0270896.s002.doc]

Supplementary Table 2 Clinical characteristics of the patients with psittacosis pneumonia

| Cases | Fever  (°C) | Cough | Fatigue | Dyspnea | Chills | Headache | Abdominal pain or diarrhea | Chest tightness or pain | Myalgia | Hemoptysis or blood-stained sputum | Vomiting or loss of appetite | Others |
| --- | --- | --- | --- | --- | --- | --- | --- | --- | --- | --- | --- | --- |
| 1# | 40.0 | Y | Y | Y | N | N | N | N | N | N | N | N |
| 2# | 39.5 | Y | N | N | Y | N | N | N | Y | N | N | Palpitation |
| 3# | 39.2 | Y | Y | N | Y | N | N | N | N | N | N | N |
| 4# | 39.9 | N | N | N | N | Y | N | N | Y | N | N | N |
| 5# | 39.1 | N | N | N | N | N | N | N | Y | N | N | N |
| 6# | Y | Y | Y | N | N | N | N | N | N | N | N | N |
| 7# | 40.0 | N | N | N | Y | N | N | N | N | N | N | N |
| 8# | 39.1 | Y | N | N | Y | Y | N | Y | Y | N | N | N |
| 9# | 39.8 | N | N | N | N | N | N | N | N | N | N | N |
| 10# | 39.6 | Y | N | Y | N | N | N | Y | Y | N | N | N |
| 11# | 39.5 | N | Y | N | Y | N | N | N | N | N | N | Dizziness, palpitation |
| 12# | 40.2 | Y | N | N | Y | N | N | N | N | N | N | N |
| 13# | 40.2 | Y | Y | N | N | N | Y | Y | N | N | N | N |
| 14# | 39.0 | N | Y | N | N | N | N | N | Y | N | Y | Large tracts of erythema |
| 15# | 40.1 | N | Y | N | N | Y | N | N | N | N | Y | Convulsion, running nose |
| 16# | 39.0 | Y | N | Y | Y | N | N | N | N | N | N | Dizziness |
| 17# | 38.7 | Y | Y | N | Y | Y | N | N | N | Y | N | N |
| 18# | Y | Y | Y | N | Y | N | N | N | N | N | N | Coma |
| 19# | 39.0 | N | N | N | Y | Y | N | N | N | N | Y | N |
| 20# | 40.0 | N | Y | Y | N | N | N | N | N | N | N | N |
| 21# | 39.6 | Y | N | Y | Y | N | Y | Y | N | N | N | N |
| 22# | Y | Y | N | Y | N | N | N | N | N | Y | N | N |
| 23# | 40.1 | Y | Y | N | Y | N | N | N | N | Y | Y | N |
| 24# | 39.5 | N | N | N | N | N | N | N | Y | N | N | N |
| 25# | 39.6 | Y | Y | N | N | Y | N | N | N | Y | N | N |
| 26* | 40.0 | Y | N | Y | N | N | N | N | N | N | Y | N |
| 27* | Y | N | Y | Y | N | N | N | Y | N | N | N | N |
| 28* | 39.0 | N | N | Y | Y | N | N | N | N | N | N | Sore throat, nasal congestion |
| 29* | 40.0 | Y | Y | Y | N | Y | N | Y | N | N | N | N |
| 30* | 40.0 | Y | Y | Y | N | N | N | N | N | N | N | N |
| 31* | 39.9 | Y | Y | Y | N | N | N | N | N | N | N | N |
| 32* | 39.3 | Y | Y | N | Y | N | N | N | N | N | N | Coma |
| 33* | 40.0 | Y | Y | Y | N | N | N | N | N | N | N | N |
| 34* | 39.0 | Y | Y | Y | N | N | N | N | N | N | N | N |
| 35* | 40.4 | Y | N | N | N | N | N | N | N | N | N | N |
| 36* | 39.0 | Y | N | Y | Y | N | N | N | N | N | N | N |
| 37* | 39.6 | Y | N | Y | N | N | N | N | N | Y | N | N |
| 38* | 39.6 | Y | Y | Y | N | Y | Y | N | Y | Y | N | N |
| 39* | 39.3 | Y | N | N | Y | N | N | N | N | N | N | N |
| 40* | 39.0 | Y | N | Y | Y | N | Y | N | N | N | N | N |
| 41* | 40.0 | Y | N | Y | N | N | N | N | N | Y | N | N |
| 42* | 38.5 | Y | Y | N | Y | N | N | N | N | N | N | Nasal congestion |
| 43* | 40.1 | N | Y | Y | N | N | Y | N | N | N | N | Coma |
| 44* | 40.0 | N | Y | Y | N | N | Y | N | N | N | N | N |
| 45* | Y | Y | N | N | N | N | N | N | N | N | N | N |
| 46* | 39.0 | Y | N | N | N | N | Y | N | N | N | N | N |
| 47* | 38.9 | N | N | N | N | Y | N | N | N | N | N | N |
| 48* | Y | Y | Y | Y | N | N | N | Y | N | N | N | N |
| 49* | Y | Y | N | N | N | Y | N | N | N | N | N | N |
| 50* | 39.5 | N | N | N | Y | N | Y | N | N | N | N | N |
| 51* | 40.2 | N | N | N | N | N | Y | Y | N | N | Y | Palpitation, sore throat |
| 52* | 40.0 | Y | Y | Y | Y | N | Y | N | N | N | N | N |

#patients with moderate psittacosis pneumonia; *patients with severe to fulminant psittacosis pneumonia

N, no; Y, yes
