# Supplementary material for: Clinical, radiological and pathological characteristics of moderate to fulminant psittacosis pneumonia
Source: PLoS One. 2022 Jul 11;17(7):e0270896. doi: 10.1371/journal.pone.0270896 (PMC9273088; doi:10.1371/journal.pone.0270896)
Supplement: S3 Table — (DOC) [file pone.0270896.s003.doc]

Supplementary Table 3 Laboratory characteristics of the patients with psittacosis pneumonia

| Cases | pH | PaO_2_ (mmHg) | PaCO_2_ (mmHg) | SO_2_ % (OI mmHg) | WBC (×109/L) | NE (%) | PLT (×109/L) | CRP (mg/L) | ESR(mm/hr) | PCT (ng/ml) | AST (U/L) | ALT (U/L) | TBIL (μmol/L) | DBIL (μmol/L) | ALB (g/L) | BUN (mmol/L) | CRE (μmol/L) | CK (U/L) | Na (mmol/L) | K (mmol/L) |
| --- | --- | --- | --- | --- | --- | --- | --- | --- | --- | --- | --- | --- | --- | --- | --- | --- | --- | --- | --- | --- |
| 1# | 7.58 | 65.5 | 29.5 | 95.2 (312) | 7.78 | 74.4 | 463 | 153.95 | 49 | NA | 51 | 74 | 16.9 | 8.3 | 36.5 | 3.44 | 54 | NA | 141.3 | 2.60 |
| 2# | 7.52 | 65.0 | 24.2 | 92.5 (310) | 6.31 | 93.3 | 323 | 219.67 | 85 | 1.77 | 67 | 61 | 16.5 | 6.8 | 25.9 | 6.12 | 82 | 321 | 144.6 | 3.10 |
| 3# | 7.54 | 70.6 | 23.7 | 96.0 (336) | 7.76 | 82.8 | 510 | 181.8 | NA | 0.95 | 89 | 84 | 7.2 | 3.0 | 25.6 | 6.12 | 73 | 117 | 141.7 | 3.27 |
| 4# | NA | NA | NA | NA | 7.08 | 71 | 237 | 90.27 | 95 | NA | 48 | 98 | 14.9 | 6.1 | 38.6 | 3.85 | 85 | 155 | 140.0 | 4.12 |
| 5# | NA | NA | NA | NA | 5.15 | 54 | 253 | 48.49 | 120 | NA | 35 | 36 | 3.9 | 1.5 | 37.2 | 3.52 | 53 | 77 | 140.6 | 4.22 |
| 6# | 7.51 | 73.7 | 29 | 94.5 (254) | 12.88 | 86.5 | 520 | 55.43 | 120 | 0.07 | 39 | 17 | 11.5 | 6.0 | 26.8 | 6.84 | 36 | 30 | 138.5 | 4.95 |
| 7# | NA | NA | NA | NA | 7.03 | 85.8 | 306 | 212.3 | 120 | 0.24 | 55 | 68 | 4.1 | 1.5 | 21.6 | 1.14 | 52 | 73 | 138.2 | 3.50 |
| 8# | NA | NA | NA | NA | 11.32 | 76.4 | 303 | NA | 69 | 0.15 | 14 | 9 | 10.6 | 3.4 | 39.4 | 2.43 | 66 | 59 | 138.2 | 3.62 |
| 9# | 7.51 | 82.1 | 25.9 | 97.2 (390) | 5.80 | 76.3 | 202 | 170.01 | 120 | 0.51 | 90 | 109 | 8.5 | 3.7 | 29.2 | 3.82 | 92 | 365 | 128.3 | 3.73 |
| 10# | 7.54 | 62.5 | 24.8 | 92.0 (298) | 7.02 | 91.4 | 327 | 259.91 | 97 | NA | 64 | 23 | 8.7 | 3.5 | 24.1 | 6.43 | 75 | 435 | 127.6 | 4.32 |
| 11# | 7.52 | 61.5 | 27.7 | 93.3 (212) | 3.39 | 85.4 | 174 | 256.02 | 120 | 0.24 | 86 | 85 | 9.5 | 2.2 | 26.8 | 6.85 | 68 | 79 | 135.2 | 2.83 |
| 12# | 7.50 | 77.7 | 24.9 | 93.1 (370) | 5.52 | 78.9 | 242 | 166.61 | 120 | 0.26 | 200 | 119 | 4.7 | 1.7 | 20.9 | 5.70 | 62 | 51 | 131.2 | 3.59 |
| 13# | 7.49 | 64.0 | 22.3 | 96.1 (305) | 5.46 | 70.9 | 245 | 28.14 | 79 | 1.04 | 110 | 102 | 21.7 | 7.3 | 32.9 | 3.71 | 64 | 97 | 135.9 | 3.92 |
| 14# | NA | NA | NA | NA | 3.97 | 87 | 174 | 293.72 | 87 | 0.48 | 92 | 76 | 52.4 | 35.4 | 26.2 | 4.13 | 83 | 454 | 128.8 | 3.50 |
| 15# | 7.52 | 56.6 | 23.2 | 90.5 (270) | 14.44 | 78 | 283 | 85.32 | 120 | 1.06 | 40 | 28 | 9.1 | 5.5 | 24.1 | 3.12 | 72 | NA | 126.0 | 3.08 |
| 16# | NA | NA | NA | NA | 4.91 | 54.5 | 373 | 75.02 | 110 | NA | 69 | 44 | 5.2 | 1.8 | 31.6 | 4.79 | 62 | 86 | 145.2 | 3.52 |
| 17# | NA | NA | NA | NA | 6.35 | 92 | 241 | 152.55 | NA | NA | 50 | 87 | 6.0 | 2.0 | 28.6 | 5.16 | 73 | 78 | 131.5 | 4.41 |
| 18# | 7.48 | 106 | 23.3 | 98.2 (321) | 3.67 | 90.8 | 119 | 6.34 | 37 | 0.13 | 66 | 47 | 8.6 | 3.7 | 25.6 | 2.26 | 69 | 75 | 143.5 | 3.61 |
| 19# | NA | NA | NA | NA | 4.33 | 93.5 | 138 | 338.15 | 104 | 1.24 | 346 | 299 | 17.2 | 12.1 | 23.7 | 4.85 | 93 | 212 | 130.2 | 3.90 |
| 20# | NA | NA | NA | NA | 6.55 | 80.5 | 206 | 278.13 | 116 | 0.53 | 28 | 16 | 4.6 | 1.8 | 33.2 | 7.54 | 90 | 156 | 136.4 | 3.66 |
| 21# | 7.43 | 65.5 | 38.5 | 93.8 (312) | 17.73 | 84.4 | 174 | 311.44 | 98 | 0.12 | 105 | 86 | 35.7 | 13.2 | 30.5 | 5.49 | 77 | 3520 | 134.5 | 4.52 |
| 22# | NA | NA | NA | NA | 10.94 | 79.9 | 199 | 7.05 | 36 | 0.86 | 35 | 26 | 6.2 | 2.5 | 33.8 | 24.12 | 971 | NA | 140.1 | 5.96 |
| 23# | 7.53 | 83 | 26.1 | 95.9 (395) | 6.64 | 89.4 | 142 | 220.62 | 110 | 0.72 | 202 | 225 | 20.7 | 9.8 | 28.7 | 8.54 | 121 | 108 | 138.6 | 4.02 |
| 24# | 7.50 | 60.9 | 32.5 | 91.8 (290) | 10.80 | 92.8 | 485 | 297.27 | 105 | 3.27 | 239 | 155 | 11.0 | 5.0 | 25.1 | 4.00 | 65 | 599 | 127.1 | 2.65 |
| 25# | 7.57 | 55.9 | 27.9 | 89.5 (266) | 4.54 | 86.2 | 437 | 248.32 | 120 | 2.43 | 1143 | 513 | 12.7 | 6.3 | 26.9 | 4.26 | 53 | 35530 | 137.7 | 3.11 |
| 26* | 7.49 | 74.6 | 25.9 | 95.9 (226) | 4.68 | 84.8 | 207 | 264.04 | 40 | 2.15 | 59 | 117 | 8.8 | 3.6 | 27.3 | 2.49 | 49 | 75 | 134.9 | 3.28 |
| 27* | 7.50 | 53.3 | 27.3 | 91.5 (162) | 18.98 | 90.2 | 330 | 216.86 | NA | 0.32 | 80 | 69 | 10.9 | 5.0 | 25.7 | 8.05 | 109 | 234 | 137.1 | 4.05 |
| 28* | 7.47 | 94.6 | 25.5 | 96.2 (286) | 3.24 | 85.7 | 95 | 152.45 | NA | 19.71 | 377 | 138 | 3.9 | 2.0 | 22.1 | 18.07 | 347 | 5023 | 133.1 | 2.85 |
| 29* | 7.45 | 81.0 | 29.1 | 94.5 (245) | 5.18 | 88.3 | 324 | 228.94 | 120 | 0.80 | 182 | 112 | 12.9 | 5.9 | 25.6 | 6.83 | 110 | 121 | 134.4 | 4.37 |
| 30* | 7.48 | 61.9 | 27.4 | 93.8 (188) | 9.84 | 96.5 | 281 | 319.75 | 96 | 18.44 | 393 | 315 | 16.2 | 9.9 | 24.3 | 8.23 | 96 | 798 | 137.9 | 3.30 |
| 31* | 7.36 | 47.9 | 40.8 | 74.6 (145) | 24.91 | 95.5 | 426 | 269.35 | NA | 0.80 | 41 | 40 | 15.1 | 6.4 | 22.6 | 6.80 | 69 | 53 | 137.7 | 3.56 |
| 32* | 7.33 | 58.2 | 32.5 | 88.2 (176) | 12.86 | 87.7 | 359 | 274.94 | NA | 35.29 | 171 | 64 | 13.0 | 5.1 | 25.3 | 30.08 | 507 | 120 | 134.7 | 4.96 |
| 33* | 7.37 | 69.5 | 34.5 | 93.8 (199) | 15.19 | 95.7 | 248 | 312 | 88 | 18.04 | 114 | 69 | 16.2 | 8.1 | 28.9 | 20.72 | 197 | 175 | 138.4 | 4.53 |
| 34* | 7.45 | 33.2 | 28.2 | 57.3 (158) | 7.05 | 95.3 | 257 | 369.77 | 82 | 60.01 | 620 | 264 | 45.1 | 22.6 | 25.7 | 21.36 | 288 | 17895 | 130.6 | 3.90 |
| 35* | 7.35 | 71.0 | 31.2 | 91.2 (244) | 3.3 | 66 | 214 | 19.72 | 28 | 0.61 | 58 | 89 | 11.8 | 7.5 | 22.1 | 5.36 | 106 | 23 | 136.7 | 3.05 |
| 36* | 7.43 | 44.8 | 25.6 | 80.0 (213) | 17.62 | 94.8 | 297 | 233.08 | 120 | 10.29 | 333 | 353 | 21.8 | 7.7 | 19.7 | 6.00 | 96 | 79 | 134.4 | 3.97 |
| 37* | 7.48 | 54.2 | 29.1 | 84.5 (258) | 6.83 | 89.7 | 208 | 270.29 | 120 | 1.56 | 610 | 369 | 18.5 | 8.5 | 25.5 | 2.95 | 65 | 351 | 136.9 | 3.56 |
| 38* | 7.51 | 54.5 | 22.1 | 85.0 (260) | 22.76 | 94.5 | 334 | 223.01 | NA | 4.72 | 286 | 192 | 51.7 | 6.9 | 23.3 | 37.26 | 223 | 275 | 132.7 | 3.21 |
| 39* | 7.46 | 73.8 | 30.5 | 96.4 (185) | 5.59 | 93.4 | 375 | 211.13 | 102 | 4.10 | 494 | 501 | 232.5 | 173.9 | 27.5 | 5.12 | 57 | 9697 | 134.1 | 3.01 |
| 40* | 7.47 | 76.9 | 29.7 | 96.6 (192) | 7.55 | 93.6 | 342 | 320.94 | 120 | 4.72 | 2009 | 846 | 24.4 | 11.2 | 24.2 | 15.23 | 132 | 148 | 142.7 | 4.19 |
| 41* | 7.50 | 53.0 | 26.1 | 86.0 (252) | 2.72 | 89.9 | 110 | 168.52 | NA | 9.75 | 184 | 73 | 14.8 | 9.5 | 17.2 | 11.21 | 120 | 196 | 126.1 | 4.31 |
| 42* | 7.50 | 80.4 | 21.6 | 97.5 (244) | 4.99 | 89.1 | 138 | 242.73 | 107 | 1.29 | 94 | 76 | 18.4 | 9.9 | 26.3 | 6.20 | 63 | 541 | 128.5 | 4.67 |
| 43* | 7.41 | 63.2 | 34.1 | 79.8 (158) | 17.36 | 95.3 | 107 | 216.77 | 32 | 18.11 | 113 | 22 | 21.3 | 16.6 | 27.5 | 23.51 | 259 | 218 | 114.3 | 3.08 |
| 44* | 7.44 | 60.1 | 31.6 | 93.3 (182) | 12.09 | 94.4 | 159 | 299.06 | 120 | 6.69 | 447 | 298 | 27.6 | 15.8 | 22.6 | 4.98 | 80 | 523 | 135.4 | 3.90 |
| 45* | 7.44 | 118 | 22.9 | 98.3 (236) | 11.76 | 95.6 | 32 | 98.21 | 110 | 24.97 | 190 | 166 | 337.5 | 261.1 | 21.6 | 18.06 | 107 | 160 | 159.9 | 2.06 |
| 46* | 7.53 | 73.3 | 28.8 | 96 (253) | 3.03 | 79.3 | 51 | 67.65 | 36 | 0.46 | 94 | 63 | 48.3 | 20.0 | 18.3 | 4.69 | 61 | 55 | 138.7 | 3.23 |
| 47* | 7.41 | 60.9 | 38.6 | 80 (152) | 18.53 | 95.1 | 222 | 279.68 | 120 | 2.17 | 196 | 221 | 30.5 | 23.3 | 23.6 | 8.24 | 79 | 30 | 138.5 | 4.34 |
| 48* | 7.52 | 52.5 | 26.5 | 76.5 (159) | 12.57 | 71.8 | 559 | 43.48 | 120 | 0.03 | 17 | 12 | 10.2 | 3.8 | 40.6 | 5.64 | 50 | 68 | 140.0 | 3.85 |
| 49* | 7.49 | 39.0 | 23.7 | 79.7 (118) | 6.57 | 86.6 | 130 | 247.58 | 120 | 1.42 | 115 | 96 | 17.7 | 10.8 | 27.4 | 3.39 | 68 | 163 | 130.6 | 3.59 |
| 50* | 7.48 | 49.7 | 29.5 | 87.7 (237) | 4.52 | 79.4 | 226 | 264.42 | NA | 1.34 | 128 | 91 | 11.4 | 5.0 | 32.9 | 7.32 | 69 | 229 | 140.3 | 4.42 |
| 51* | 7.58 | 50.0 | 16.6 | 89.3 (143) | 14.9 | 93 | 121 | 232.88 | 57 | 6.94 | 50 | 29 | 10.4 | 4.6 | 22.0 | 5.37 | 59 | 42 | 138.6 | 2.86 |
| 52* | 7.30 | 64.4 | 50.9 | 91.1 (64) | 13.53 | 88.7 | 425 | 137.67 | NA | 1.61 | 407 | 169 | 42.3 | 15.2 | 25.6 | 6.76 | 46 | NA | 140.0 | 3.02 |

#patients with moderate psittacosis pneumonia; *patients with severe to fulminant psittacosis pneumonia

ALB, albumin; ALT, alanine aminotransferase; AST, aspartate aminotransferase; BUN, blood urea nitrogen; CK, creatine kinase; CRE, creatinine; CRP, C-reactive protein; DBIL, direct bilirubin; ESR, erythrocyte sedimentation rate; K, potassium; Na, natrium; NA, not available; NE, neutrophil ratio; OI, oxygenation index; PaCO2, arterial partial pressure of carbon dioxide; PaO2, arterial partial pressure of oxygen; PCT, procalcitonin; pH, pondus hydrogenii; PLT, platelet; TBIL, total bilirubin; WBC, white blood cell counts; SO2, oxygen saturation
