# Supplementary material for: Clinical, radiological and pathological characteristics of moderate to fulminant psittacosis pneumonia
Source: PLoS One. 2022 Jul 11;17(7):e0270896. doi: 10.1371/journal.pone.0270896 (PMC9273088; doi:10.1371/journal.pone.0270896)
Supplement: S4 Table — (DOC) [file pone.0270896.s004.doc]

Supplementary Table 4 Radiologic characteristics, treatment and outcomes of the patients with psittacosis pneumonia

| Cases | Lung lesions | Diagnosis (days) | Start time (days) | ICU staying  (days) | Length of stay  (days) | Noninvasive ventilation  (days) | Invasive ventilation  (days) | ECMO  (days) | Hemofiltration  (times) | Antibiotic therapy | Complications | Outcome |
| --- | --- | --- | --- | --- | --- | --- | --- | --- | --- | --- | --- | --- |
| 1# | RML, RUL!; RPE | 3 | 23 | NA | 7 | NA | NA | NA | NA | DOX + MOX | HD, HK | REC |
| 2# | RLL! to BL | 4 | 8 | NA | 13 | NA | NA | NA | NA | PIP to DOX | HD, HK, HP | REC |
| 3# | LUL! | 2 | 8 | NA | 8 | NA | NA | NA | NA | PIP to DOX | HD, HK, HP | REC |
| 4# | LLL, LUL! | 3 | 9 | NA | 5 | NA | NA | NA | NA | PIP to DOX | HD | REC |
| 5# | RUL! | 3 | 6 | NA | 5 | NA | NA | NA | NA | MOX | NA | REC |
| 6# | LLL, RLL | 2 | 32 | NA | 10 | NA | NA | NA | NA | MER to DOX | HP, RF | REC |
| 7# | RUL! | 4 | 10 | NA | 12 | NA | NA | NA | NA | MOX to DOX | HD, HP | REC |
| 8# | RUL! | 3 | 7 | NA | 6 | NA | NA | NA | NA | MOX | NA | REC |
| 9# | RLL! | 2 | 8 | NA | 15 | NA | NA | NA | NA | DOX | HD, HN, HP | REC |
| 10# | RML, RUL!; RPE | 2 | 9 | NA | 7 | NA | NA | NA | NA | PIP to MOX | HP, RF | REC |
| 11# | RLL! to BL; BPE | 3 | 8 | NA | 13 | NA | NA | NA | NA | MOX | HD, HK, HP, RF | REC |
| 12# | RUL!; RPE | 2 | 6 | NA | 6 | NA | NA | NA | NA | MOX | HD, HK, HP | REC |
| 13# | RUL! to BL; RPE | 4 | 13 | NA | 8 | NA | NA | NA | NA | MER to DOX | HD, HP, RF | REC |
| 14# | LLL!; LPE | 3 | 15 | NA | 13 | NA | NA | NA | NA | PIP to DOX | HD, HP | REC |
| 15# | RML to RUL! | 3 | 14 | NA | 10 | NA | NA | NA | NA | PIP to DOX | HK, HN, HP, RR | REC |
| 16# | RLL! | 3 | 13 | NA | 5 | NA | NA | NA | NA | PIP to DOX | HD, HP | REC |
| 17# | RML, RUL!; RPE | 3 | 7 | NA | 7 | NA | NA | NA | NA | MOX | HD, HP, HN | REC |
| 18# | LLL, RLL! | 9 | 16 | NA | 13 | NA | NA | NA | NA | MER to DOX | HD, HP | REC |
| 19# | LLL! | 5 | 5 | NA | 7 | NA | NA | NA | NA | DOX | HD, HP | REC |
| 20# | LUL! to BL | 6 | 3 | NA | 8 | NA | NA | NA | NA | CEF to DOX | HP | REC |
| 21# | LLL! | 2 | 6 | NA | 9 | NA | NA | NA | NA | MOX to DOX | HD, HP, RH | REC |
| 22# | RLL! to BL | 6 | 16 | NA | 8 | NA | NA | NA | NA | PIP to DOX | HP | REC |
| 23# | LUL! to BL | 2 | 17 | NA | 12 | NA | NA | NA | NA | CET to DOX | HD, HP | REC |
| 24# | RLL! | 6 | 10 | 5 | 14 | NA | NA | NA | NA | PIP + MOX to DOX | HD, HK, HP, RF | REC |
| 25# | RLL! | 3 | 7 | 6 | 15 | NA | NA | NA | NA | DOX | HD, HK, HP, RH, RF | REC |
| 26* | LUL, RLL!, RUL | 3 | 11 | 7 | 12 | 6 | NA | NA | NA | DOX | HD, HK, HP, RF | REC |
| 27* | RUL! to BL; BPE | 3 | 7 | 6 | 11 | 6 | NA | NA | NA | CEF to DOX | HD, HP, RF | REC |
| 28* | LLL, LUL!; LPE | 2 | 7 | 3 | 3 | NA | NA | NA | NA | DOX | HP, HK, MODS (HD + RD + RF), RH | ABA |
| 29* | RLL!; RPE | 2 | 8 | 5 | 14 | 2 | NA | NA | NA | MOX to DOX | HD, HP, RF | REC |
| 30* | LLL! to BL; LPE | 2 | 7 | 12 | 19 | 6 | NA | NA | NA | DOX | HD, HK, HP, RF | REC |
| 31* | RLL, LLL | 2 | 9 | 10 | 16 | 12 | NA | NA | NA | PIP to DOX | HP, RF | REC |
| 32* | RLL! to BL | 3 | 7 | 17 | 24 | 5 | 11 | NA | NA | DOX + MER | HP, MODS (HD + RD + RF) | REC |
| 33* | RUL! to BL; BPE | 3 | 7 | 12 | 26 | VOT 14 | 9 | NA | NA | MOX + MER | GH, HP, MODS (HD + RD + RF) | REC |
| 34* | RLL! to BL | 4 | 17 | 17 | 30 | 5 | 12 | NA | 2 | DOX + CET | HP, MODS (HD + RD + RF), PE, RH | REC |
| 35* | LLL, RLL; BPE | 1 | 8 | 5 | 13 | NA | NA | NA | NA | MOX + CET | HD, HK, HP, RF | REC |
| 36* | LLL, RLL; BPE | 5 | 6 | 9 | 20 | 1 | 6 | NA | NA | MOX to DOX | HD, HP, RF | REC |
| 37* | LLL!, RLL, LUL, LPE | 2 | 7 | 10 | 17 | 4 | NA | NA | NA | MOX to DOX | HD, HP, RF | REC |
| 38* | RLL!, RUL, LUL; RPE | 4 | 8 | 10 | 19 | 1 | 8 | NA | NA | MOX + DOX | HK, HN, HP, MODS (HD + RD + RF), PE | REC |
| 39* | LLL, RLL!; RPE | 2 | 7 | 9 | 17 | 2 | 5 | NA | NA | MOX to DOX | HD, HP, RF, RH | REC |
| 40* | LLL!, RUL, RLL; BPE | 5 | 10 | 13 | 21 | 2 | 16 | NA | 10 | PIP to MOX + DOX | MODS (HD + RD + RF), HP, PE, SS | ABA |
| 41* | RUL! to BL; BPE | 4 | 14 | 4 | 18 | 1 | NA | NA | NA | PIP to DOX | MODS (HD + RD + RF), HP, PE | REC |
| 42* | LUL!; LPE | 6 | 8 | 4 | 15 | 2 | NA | NA | NA | CET + MOX to DOX | HD, HP, RF | REC |
| 43* | RLL!, RUL to BL | 2 | 6 | 3 | 3 | 1 | 3 | 1 | NA | MER to DOX | HD, HP, RF, SS | ABA |
| 44* | RLL! to BL; BPE | 2 | 7 | 15 | 7 | 1 | NA | NA | NA | MER + MOX to DOX | HD, HP, RF, SS | REC |
| 45* | LLL, RLL; BPE | 2 | 7 | 12 | 15 | NA | 11 | 8 | NA | MER to PIP + DOX | HD, HK, HP, RF, PE, SS | REC |
| 46* | LLL, RLL | 3 | 6 | 7 | 11 | NA | NA | NA | NA | MOX to DOX | HD, HK, HP, RF | REC |
| 47* | RUL! to BL; BPE | 2 | 5 | 9 | 15 | 2 | 7 | 5 | NA | MER to DOX + VAN | HD, HP, PE, RF, SS | REC |
| 48* | LUL!; BPE | 2 | 12 | 14 | 28 | NA | 10 | NA | NA | MOX + CET | RF | REC |
| 49* | LUL! to BL; LPE | 5 | 8 | 7 | 17 | 4 | NA | NA | NA | PIP + DOX | HD, HN, HP, RF | REC |
| 50* | RLL!, RUL; RPE | 1 | 11 | 5 | 12 | 4 | NA | NA | NA | PIP + DOX | HD, HP, RF | REC |
| 51* | RLL! to BL; BPE | 1 | 8 | 9 | 23 | 1 | 6 | NA | NA | PIP + DOX | HK, HP, RF | REC |
| 52* | LLL, RLL | 1 | 18 | 55 | 55 | 7 | 32 | 18 | NA | MER + DOX | HK, HP, RF | REC |

#patients with moderate psittacosis pneumonia; *patients with severe to fulminant psittacosis pneumonia; ! the most severely involved lobe

ABA, abandoning treatment; BL, both lungs; BPE, bilateral pleural effusion; CEF, ceftriaxone; CET, cefperazone- tazobactam; Diagnosis, the diagnosis time after hospitalization; DOX, doxycycline; ECMO, extracorporeal membrane oxygenation; GH, gastrointestinal hemorrhage; HD, hepatic dysfunction; HK, hypokalemia; HN, hyponatremia; HP, hypoproteinemia; ICU, intensive care unit; LLL, left lower lobe; LPE, left pleural effusion; LUL, left upper lobe; MER, meropenem; MOX, moxifloxacin; PE, pericardial effusion; PIP, piperacillin-tazobactam; RD, renal dysfunction; REC, recovery; RF, respiratory failure; RH, rhabdomyolysis; RL, right lung; RLL, right lower Lobe; RML, right middle lobe, RPE, right pleural effusion; RUL, right upper Lobe; SS, septic shock; Start time, the start time of specific treatment; VAN, vancomycin; VOT, Venturi oxygen therapy
