# Supplementary figures and images for: Clinical, radiological and pathological characteristics of moderate to fulminant psittacosis pneumonia
Source: PLoS One. 2022 Jul 11;17(7):e0270896. doi: 10.1371/journal.pone.0270896 (PMC9273088; doi:10.1371/journal.pone.0270896)

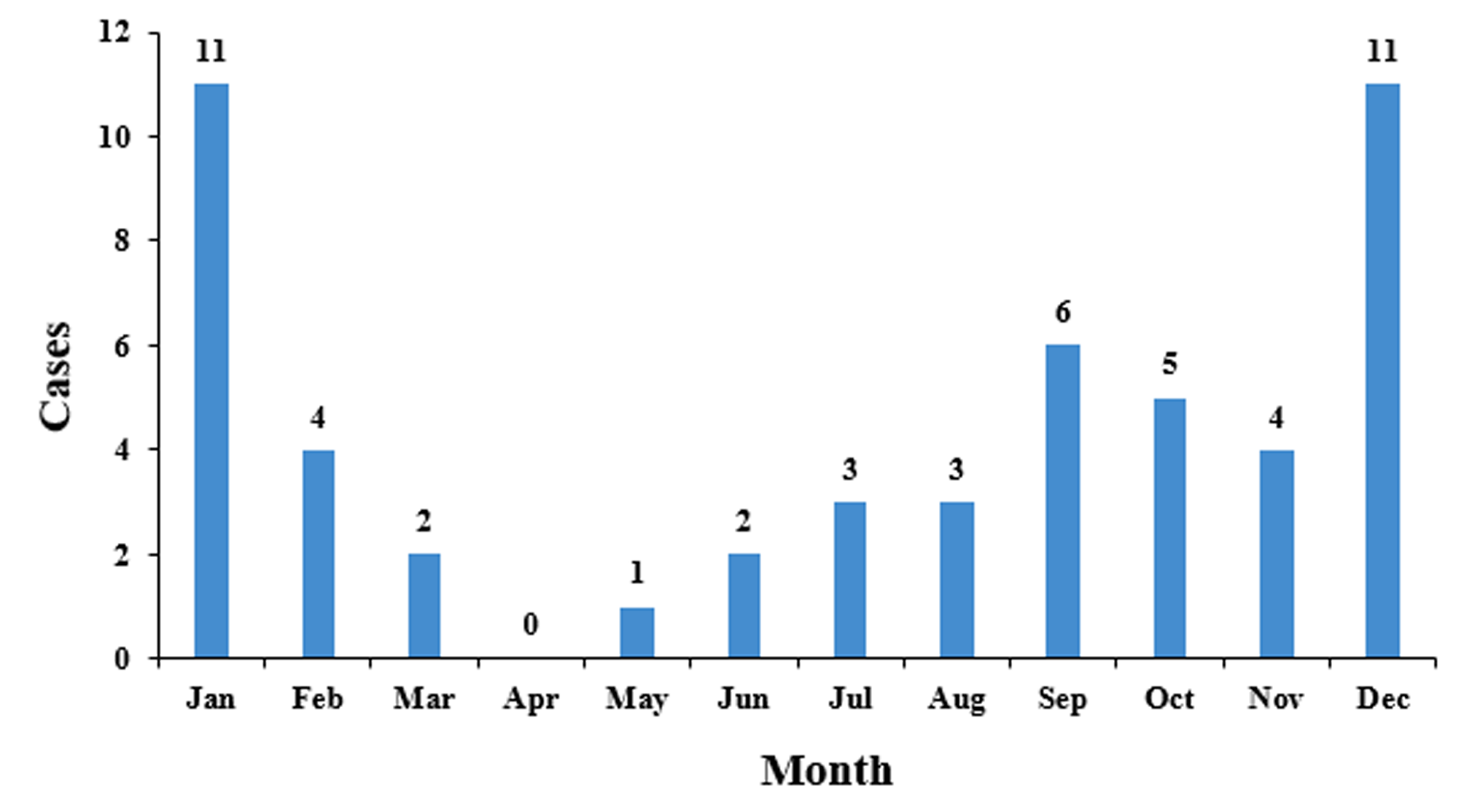

Supplement: S1 Fig — (TIF) [file pone.0270896.s005.tif]

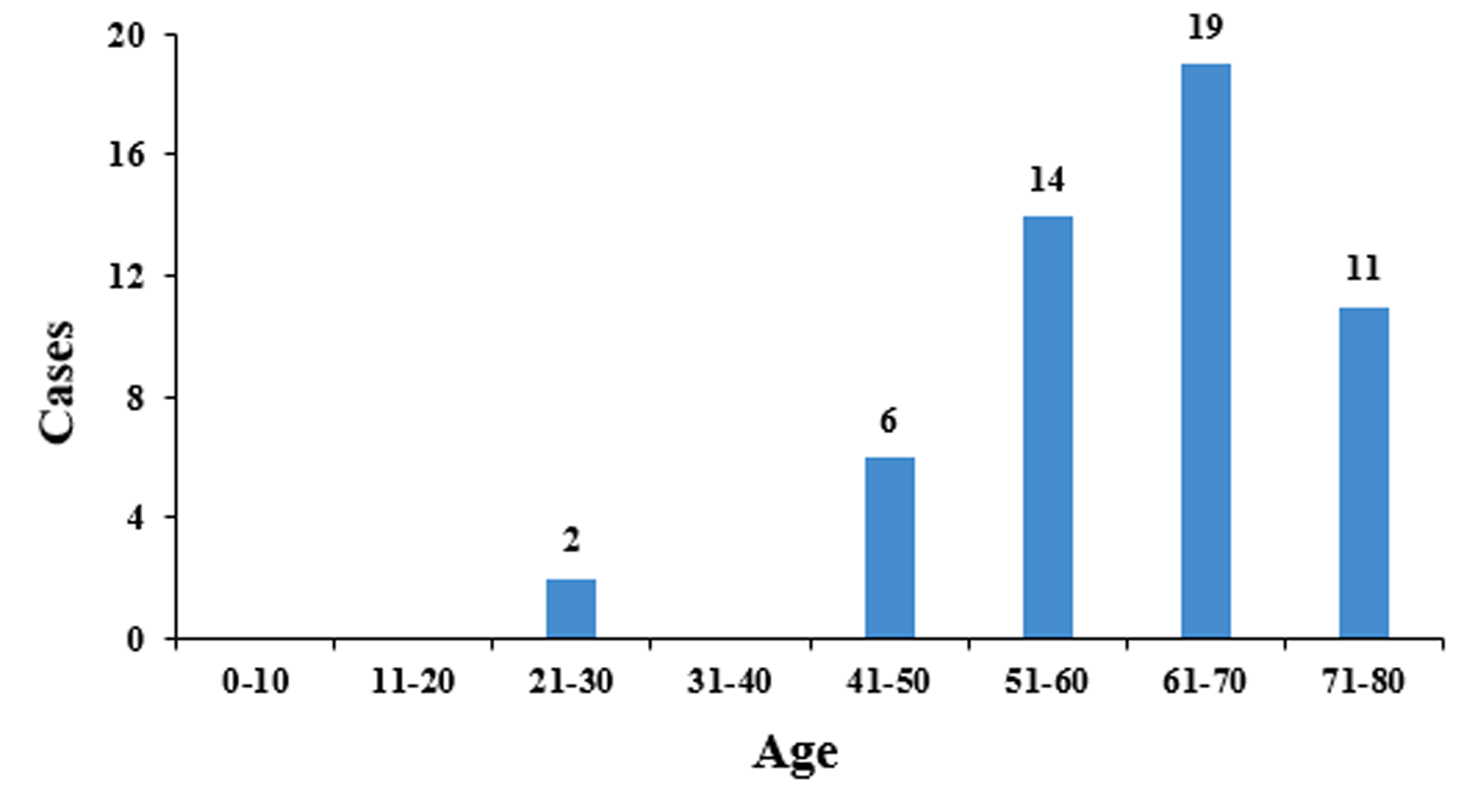

Supplement: S2 Fig — (TIF) [file pone.0270896.s006.tif]
